# Trends in mortality due to ischemic heart diseases among patients with Alzheimer's disease in the United States from 1999 to 2020

**DOI:** 10.1016/j.ijcrp.2025.200390

**Published:** 2025-03-07

**Authors:** Muzamil Akhtar, Hanzala Ahmed Farooqi, Rayyan Nabi, Javed Iqbal, Sabahat Ul Ain Munir Abbasi, Muhammad Rashid, Syed Khurram Mushtaq Gardezi, David P. Ripley, Raheel Ahmed

**Affiliations:** a Gujranwala Medical College, Gujranwala, Pakistan; b Islamic International Medical College, Riphah International University, Rawalpindi, Pakistan; c Hamad Medical Corporation, Doha, Qatar; d Allama Iqbal Medical College, Lahore, Pakistan; e National Institute of Health Research, Keele University, Keele, UK; f Department of Cardiology, Sheikh Shakhbout Medical City, Abu Dhabi, United Arab Emirates; g College of Medicine and Health Sciences, Khalifa University, Abu Dhabi, United Arab Emirates; h Northumbria Healthcare NHS Foundation Trust, Northumberland, UK; i National Heart and Lung Institute, Imperial College London, UK

**Keywords:** Alzheimer's disease, Cardiovascular disease, CDC wonder, Ischemic heart disease

## Abstract

**Background:**

Ischemic heart diseases (IHD) and Alzheimer's Disease (AD) significantly contribute to mortality in aging population. Understanding mortality trends where these conditions overlap is crucial for developing targeted interventions for vulnerable populations.

**Methods:**

We analyzed CDC WONDER mortality data from 1999 to 2020 for individuals aged ≥45 years. IHD and AD mortality were identified using ICD-10 codes I20-I25 and G30, respectively. Age-adjusted mortality rates (AAMR) per 100,000 were calculated, and trends were analyzed by gender, race, region, place of death and state. Joinpoint regression was used to calculate annual percentage changes (APC) with 95 % confidence intervals (CI).

**Results:**

A total of 171,080 deaths were attributed to IHD in individuals with AD from 1999 to 2020. The AAMR decreased from 10.6 in 1999 to 4.1 in 2020, with a significant decline between 2004 and 2014 (APC: −7.73; 95 % CI: −8.42 to −7.24). Females exhibited higher overall AAMR compared to males (Females: 6.8 vs. Males: 6.4). Individuals of Non-Hispanic (NH) White ancestry had the highest AAMR (6.8), followed by those of NH Black (6.5) and Hispanic ancestry (5.9). The West region reported the highest AAMR at 7.9, while the Midwest had the lowest at 6.3. Oklahoma recorded the highest state-level AAMR (10.9), while Utah had the lowest (3.2).

**Conclusions:**

IHD mortality in individuals with AD declined significantly, with disparities by gender, race, and geography. These findings underscore the need for tailored public health approaches to address the evolving burden of IHD in AD patients.

## Graphical abstract

**Figure:**
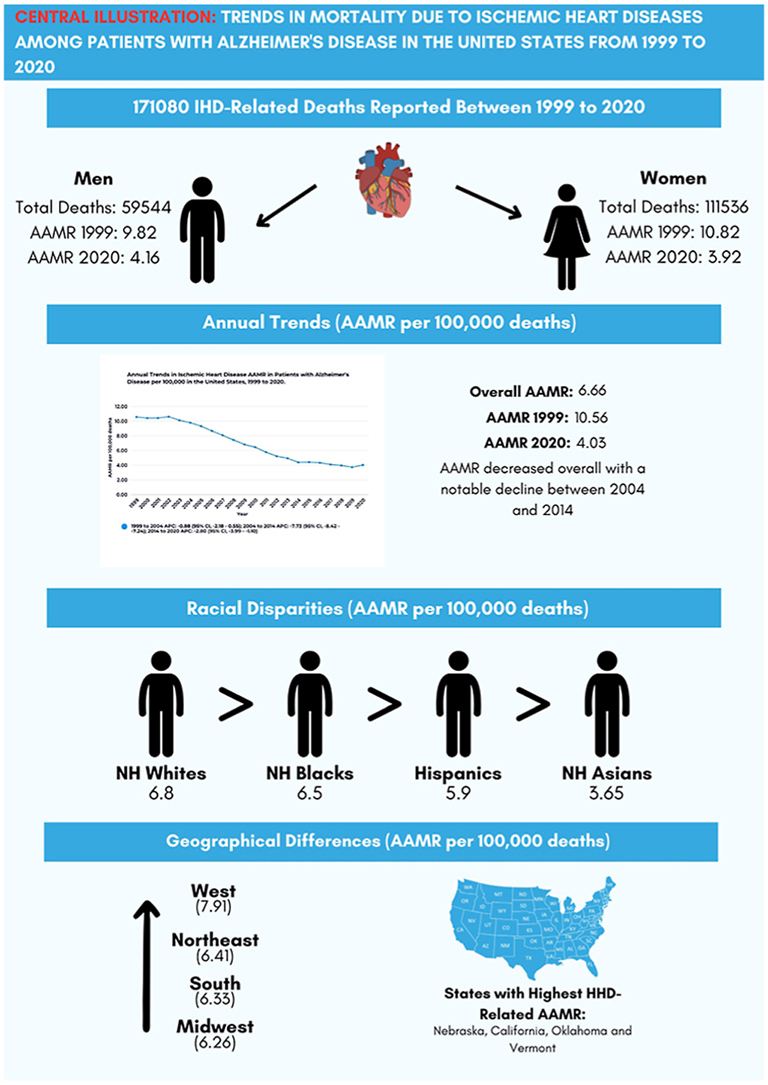

## List of abbreviations

**AD**: Alzheimer's Disease
**IHD**: Ischemic Heart Diseases
**AAMR**: Age-Adjusted Mortality Rate
**APC**: Annual Percentage Change
**AAPC**: Average Annual Percentage Change
**CDC WONDER**: Centers for Disease Control and Prevention's Wide‐Ranging Online Data for Epidemiologic Research

## 1 Introduction

Studies show that approximately 6.7 million people in the United States (US) suffer from Alzheimer's disease (AD). AD significantly impacts public health, ranking as the sixth leading cause of death in the US in 2019. Moreover, the total expenditure for healthcare of AD patients older than 65 years was $345 billion in 2023. This highlights the massive public and economic impact that AD has on healthcare [1]. Studies have shown that cardiovascular disease (CVD) and its associated risk factors are often linked to the long-term development of Alzheimer's disease [2]. Among the various types of CVDs that exist, ischemic heart disease (IHD) is a significant cause of mortality among patients. Data reported a total of 8.9 million cases of IHD in the US in 2019 [3].

Ranganathan et al. analyzed the mortality trends in cardiovascular mortality among patients with AD in the US population from 1999 to 2020 [4]. Among the different types of cardiovascular diseases, the authors listed IHD as a major contributor of mortality in patients with AD. However, the analysis was limited to overall cardiovascular disease (CVD) mortality in AD patients and did not specifically focus on mortality trends related to IHD in AD across different subgroups. Therefore, to better understand this gap in literature, we conducted a 22-retrospective analysis of the CDC Wonder database to analyse mortality trends specific to IHD in patients with AD in the US population from 1999 to 2020. Understanding the trends in these diseases can help prioritize public health interventions and policies aimed at prevention and management. In addition to improving healthcare planning and resource allocation, it will also give clinicians a better understanding of IHD related mortality in AD patients across different demographic subgroups, allowing them to tailor their treatment plans accordingly.

## 2 Methods

### 2.1 Study setting and population

Data on IHD and AD related mortality in the US population aged ≥45 from 1999 to 2020 was retrieved from the CDC Wonder (Centers for Disease Control and Prevention Wide-Ranging Online Data for Epidemiologic Research) database [5]. The Multiple Cause-of-Death Public Use records were studied to identify deaths where IHD was listed as the underlying cause of death and AD was listed as a contributing cause of death. The CDC WONDER is a publicly available online database that contains public health data, including mortality data, since the year 1999. Relevant data from death certificates was extracted using the following International Classification of Diseases, Tenth Revision, Clinical Modification (ICD-10-CM) codes: G30 and I25-I29 for Alzheimer's disease and IHD, respectively. Data was retrieved for all age groups, however because there were few cases for those under the age of 45, they were excluded. The study followed the STROBE (Strengthening the Reporting of Observational Studies in Epidemiology) reporting guidelines and did not require approval from the local institutional review board [6].

### 2.2 Data abstraction

Data on year, gender, region, race, place of death and US states was extracted. Place of death was categorised as medical facilities, home, hospice, and nursing home/long-term care facilities. Races included were Non-Hispanic (NH) American Indian or Alaska Native, NH Asian or Pacific Islander, NH Black or African American, White and Hispanic or Latino. NH American Indians were excluded from analysis due to unreliable data. Regions were classified into into Northeast, Midwest, South and North based on census bureau definitions.

### 2.3 Statistical analysis

Crude mortality rates per 100,000 people were calculated by dividing the number of IHD and AD related deaths by the corresponding U.S. population of that year. The age-adjusted mortality rates (AAMRs) per 100,000 people were determined by standardizing IHD and AD related deaths to the U.S. population of the year 2000^7^. This method allows for the calculation of a weighted mean, preventing biased comparisons of mortality rates across diverse populations or time periods. We used the Joinpoint Regression Program 5.0.2. to conduct a regression analysis that helped us calculate the annual percentage changes (APCs) and average annual percentage changes (AAPCs) in AAMR, along with 95 % CI. Using Jointpoint helps identify significant shifts in AAMR over time by applying log-linear regression models, thereby accounting for periods with temporal variations. The Monte Carlo permutation test was applied to compute AAMRs at the identified segments by joining the points [7]. APCs were interpreted as increasing or decreased based on whether the slope indicating the change in mortality was significantly different from zero, confirmed through two-tailed t-tests. A P value equal to or below 0.05 indicates statistical significance.

## 3 Results

Between 1999 and 2020, a total of 171,080 deaths were recorded due to IHD among individuals aged 45 years and older who had AD. Of these, 111,536 deaths occurred in females and 59,544 in males (Supplementary Table 1). Most deaths took place in nursing homes or long-term care facilities (54.9 %), followed by medical facilities (19.34 %), and at the decedent's homes (18.9 %).

### 3.1 Annual trends

**Fig. 1:**
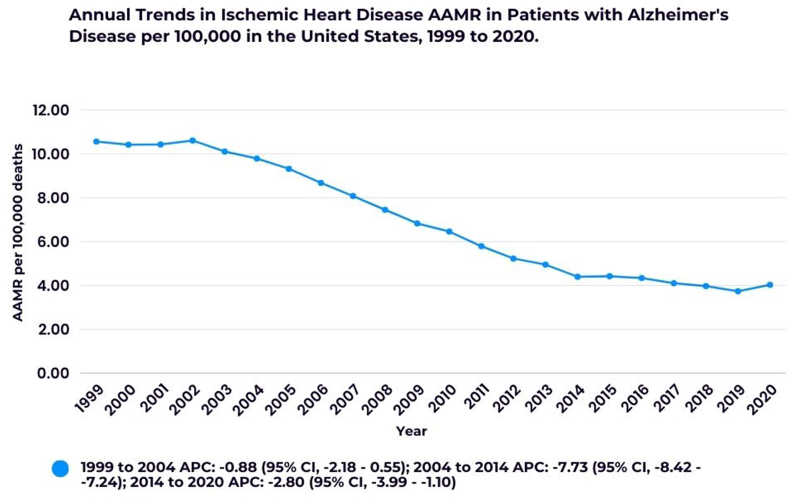
Annual trends in Ischemic Heart Disease AAMR in patients with Alzheimer's Disease. ∗Indicates that the Annual Percent Change (APC) is significantly different from zero at the alpha = 0.05 level.

The AAMR decreased steadily over the study period, dropping from 10.56 (95 % CI, 10.35–10.77) in 1999 to 4.03 (95 % CI, 3.93–4.14) in 2020 with an AAPC of −4.74 (95 % CI, −4.98 to −4.51). From 1999 to 2004, the AAMR showed a slight decline (APC: −0.88; 95 % CI, −2.18 - 0.55), followed by a sharper drop between 2004 and 2014 (APC: −7.73; 95 % CI, −8.42 to −7.24). A more moderate decrease was observed from 2014 to 2020 (APC: −2.80; 95 % CI, −3.99 to −1.10) **(**Supplementary Table 2; Fig. 1).

### 3.2 Gender-stratified trends

Over the course of the study, females consistently had a higher AAMR than males. The AAMR for females was 6.79 (95 % CI, 6.75–6.83), compared to 6.36 (95 % CI, 6.31–6.42) for males.

Among females, the AAMR decreased steadily throughout the study period. A slight decrease was seen from 1999 to 2003 (APC: −0.40; 95 % CI, −1.50 - 1.07), followed by significant declines from 2003 to 2008 (APC: −6.30; 95 % CI, −7.21 to −4.83) and from 2008 to 2014 (APC: −8.97; 95 % CI, −10.71 to −8.27). A more moderate decline was observed from 2014 to 2020 (APC: −2.54; 95 % CI, −3.43 to −1.39).

**Fig. 2:**
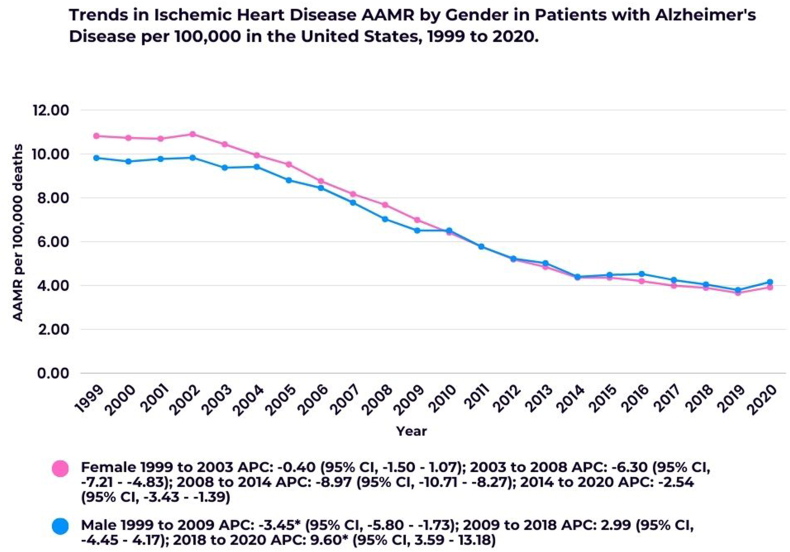
Trends in Ischemic Heart Disease AAMR in patients with Alzheimer's Disease stratified by sex. ∗Indicates that the Annual Percent Change (APC) is significantly different from zero at the alpha = 0.05 level.

In males, the AAMR decreased slightly from 1999 to 2004 (APC: −0.53; 95 % CI, −2.10 - 1.69), followed by a significant reduction from 2004 to 2014 (APC: −7.05; 95 % CI, −8.34 to −6.40). A moderate decrease was also seen between 2014 and 2020 (APC: −2.60; 95 % CI, −4.12 - 0.26) (Supplementary Table 3; Fig. 2).

### 3.3 Race-stratified trends

When stratified by race and ethnicity, the individuals of NH White ancestry recorded the highest AAMR at 6.84 (95 % CI, 6.81–6.88), followed by those of NH Black (6.49; 95 % CI, 6.38–6.61), Hispanic (5.93; 95 % CI, 5.81–6.05), and NH Asian ancestry at (3.65; 95 % CI, 3.52–3.78).

In NH White population, the AAMR remained relatively stable between 1999 and 2002. However, a significant decrease was observed from 2002 to 2006 (APC: −4.66; 95 % CI, −6.55 to −3.17), and from 2006 to 2014 (APC: −8.20; 95 % CI, −9.27 to −7.66). A more moderate decline continued from 2014 to 2020 (APC: −2.32; 95 % CI, −3.32 to −1.04).

Among NH Black individuals, the AAMR was stable from 1999 to 2005, followed by a notable decrease between 2005 and 2014 (APC: −9.07; 95 % CI, −10.98 to −8.05). A slight decline was observed from 2014 to 2020 (APC: −2.30; 95 % CI, −4.31 - 1.16).

In the Hispanic population, a significant increase in AAMR was observed from 1999 to 2004 (APC: 3.54; 95 % CI, 0.52–8.33). This was followed by a decline from 2004 to 2018 (APC: −5.96; 95 % CI, −7.80 to −5.41), but an increase was noted again from 2018 to 2020 (APC: 3.31; 95 % CI, −4.63 - 7.91).

**Fig. 3:**
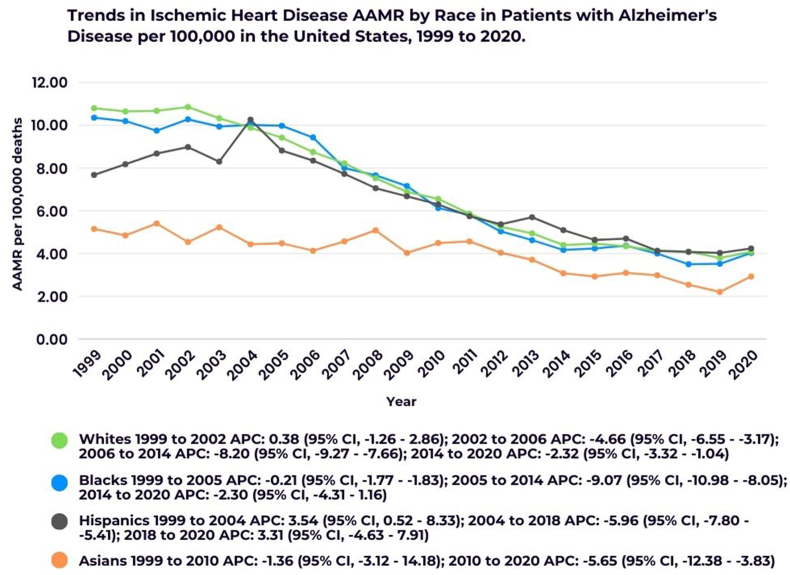
Trends in Ischemic Heart Disease AAMR in patients with Alzheimer's Disease stratified by race. ∗Indicates that the Annual Percent Change (APC) is significantly different from zero at the alpha = 0.05 level.

For NH Asians, a slight decline in AAMR was recorded from 1999 to 2010 (APC: −1.36; 95 % CI, −3.12 - 14.18), followed by a significant decrease from 2010 to 2020 (APC: −5.65; 95 % CI, −12.38 to −3.83) (Supplementary Table 4; Fig. 3).

### 3.4 State and region-based differences

**Fig. 4:**
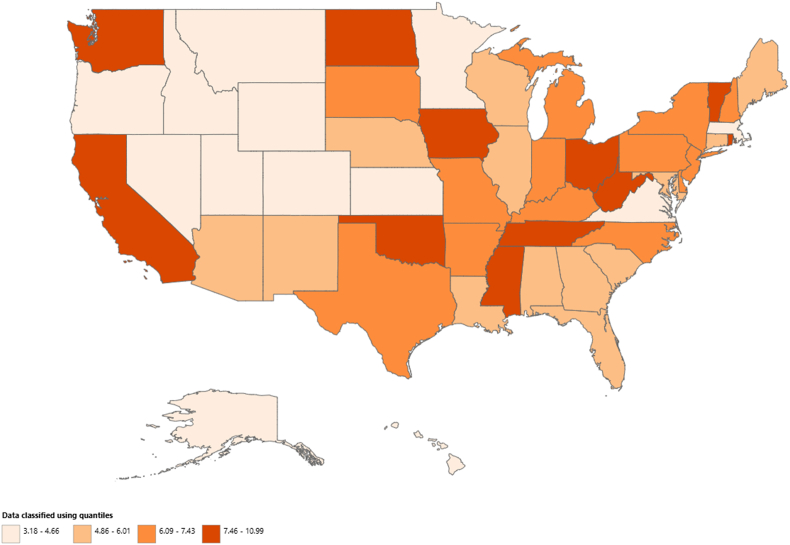
State-wise map highlighting the states with highest mortality rates in the United States from 1999 to 2020.

Age-adjusted mortality rates varied significantly across states, ranging from 3.18 (95 % CI, 2.89–3.47) in Utah to 10.99 (95 % CI, 10.61–11.36) in Oklahoma. States in the highest AAMR percentile included Oklahoma, California, Tennessee, and West Virginia, with AAMRs approximately three times higher than those in the lowest percentile states, such as Utah, Alaska, Minnesota, and Montana. California reported the highest percentage of total deaths (16.98 %), followed by New York (7.21 %) and Texas (6.18 %) (Supplementary Table 6; Fig. 4). Fig. 4 shows a map of these state-level differences.

**Fig. 5:**
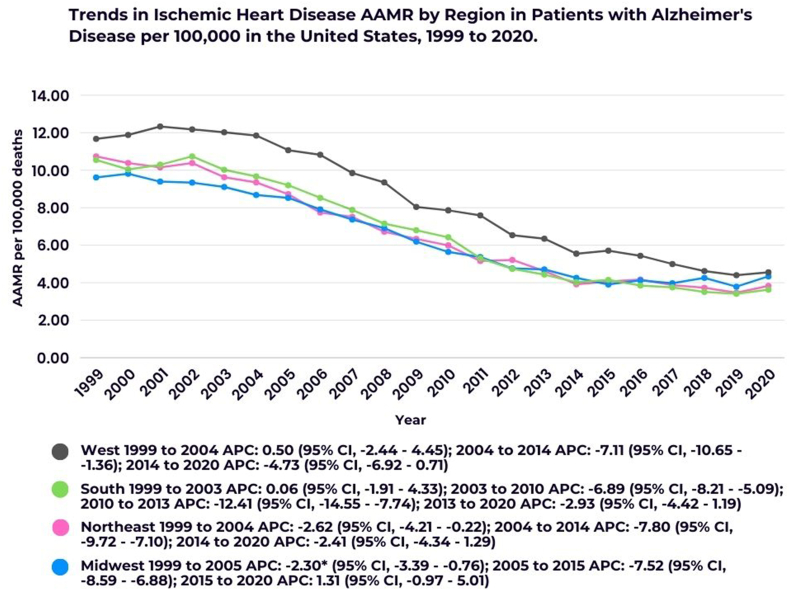
Trends in Ischemic Heart Disease AAMR in patients with Alzheimer's Disease stratified by regions. ∗Indicates that the Annual Percent Change (APC) is significantly different from zero at the alpha = 0.05 level.

Regionally, the West exhibited the highest AAMR at 7.91 (95 % CI, 7.83–7.98), with a significant decline from 2004 to 2014 (APC: −7.11; 95 % CI, −10.65 to −1.36), followed by a further decrease from 2014 to 2020 (APC: −4.73; 95 % CI, −6.92 - 0.71). The Northeast had the second-highest AAMR at 6.41 (95 % CI, 6.35–6.48), showing a substantial decline from 2004 to 2014 (APC: −7.80; 95 % CI, −9.72 to −7.10). The South reported an AAMR of 6.33 (95 % CI, 6.27–6.38), with a notable reduction from 2003 to 2010 (APC: −6.89; 95 % CI, −8.21 to −5.09) and a sharp decline between 2010 and 2013 (APC: −12.41; 95 % CI, −14.55 to −7.74). The Midwest had the lowest AAMR at 6.26 (95 % CI, 6.20–6.33), with a decline from 2005 to 2015 (APC: −7.52; 95 % CI, −8.59 to −6.88), followed by a slight increase from 2015 to 2020 (APC: 1.31; 95 % CI, −0.97 - 5.01) (Supplementary Table 5; Fig. 5).

## 4 Discussion

Our research reveals a significant reduction in the age-adjusted mortality rates (AAMR) linked to Alzheimer's disease and ischemic heart disease (IHD) in the United States between 1999 and 2020. This decline may result from various factors, such as advancements in medical treatment, heightened awareness of these illnesses, and enhanced access to healthcare. For example, the advent of new pharmacological treatments for Alzheimer's, like cholinesterase inhibitors and memantine, has been shown to slow cognitive deterioration and improve patients' quality of life, which may help lower mortality rates associated with the disease [8,9]. While these advancements enhance patient care, they have not significantly impacted overall AD mortality rates, as indicated by recent data [10]. In addition, public health efforts aimed at mitigating cardiovascular risk factors, including hypertension and diabetes, are likely instrumental in reducing IHD mortality, given the strong connection between these conditions and heart disease [11,12].

The decline in AAMR may also reflect a wider shift in healthcare toward preventive measures and early intervention. For instance, increased screening for cognitive decline and heart conditions enables earlier diagnoses and management, leading to improved outcomes [8]. Furthermore, employing multidisciplinary strategies to manage comorbidities, especially among older adults, has been shown to enhance patient care and reduce mortality rates [13,14]. This is particularly pertinent due to the high incidence of coexisting conditions in individuals with Alzheimer's and IHD, which complicates treatment and elevates mortality risk [13]. Additionally, demographic changes in the U.S. population, particularly an aging demographic with a stronger focus on geriatric care, may have played a role in the decline of AAMR. As the population ages, there is a greater emphasis on effectively managing chronic illnesses, potentially resulting in better survival rates for older adults with Alzheimer's and heart disease [15]. Moreover, the rise in availability of home health services and community support initiatives may offer enhanced assistance for patients and their families, further improving health outcomes [16]. However, it is important to note that despite the decline in IHD mortality among AD patients observed in our study, the overall AAMR for Alzheimer's disease increased by 97.4 % from 1999 to 2020, as reported by Khan et al. [10]. This contrasting trend suggests that other factors may be contributing to the rising overall AD mortality, highlighting the complexity of disease management in this population.

In our findings, we also noted that females consistently exhibited a higher AAMR than males throughout the study period. This gender disparity in mortality rates can be attributed to several biological and sociocultural factors. Research indicates that women are more likely to develop Alzheimer's disease and experience more severe symptoms compared to men, which may contribute to higher mortality rates [17,18]. Additionally, hormonal differences, particularly the decline in estrogen levels post-menopause, have been implicated in the increased risk of Alzheimer's disease among women [19,20]. Furthermore, women often live longer than men, which increases their likelihood of developing age-related diseases, including Alzheimer's and heart disease [21]. The consistent decline in AAMR for both genders suggests that while women may have a higher baseline risk, improvements in healthcare and treatment options are benefiting both sexes. The decline in mortality rates across genders may reflect the effectiveness of public health campaigns aimed at educating individuals about the risks associated with Alzheimer's disease and heart disease, as well as the importance of lifestyle modifications in managing these conditions^10,11^. Furthermore, the recognition of gender differences in the presentation and progression of Alzheimer's disease has led to more tailored approaches in treatment and care, which may also contribute to the observed decline in mortality rates [15,18,22].

The findings of our study also indicate that the overall age-adjusted mortality rates (AAMR) due to Alzheimer's disease and cardiovascular diseases from 1999 to 2020 were highest in the white population, followed by the African American population. This demographic disparity in mortality rates can be attributed to several interrelated factors, including genetic predispositions, socioeconomic status, and lifestyle choices. Research has shown that genetic factors, such as variations in inflammatory pathways, can significantly influence susceptibility to both Alzheimer's disease and cardiovascular conditions, including heart disease [23]. Moreover, socioeconomic status has been linked to health outcomes, with lower socioeconomic groups often experiencing higher rates of chronic diseases and associated mortality [24].

Additionally, lifestyle factors such as diet, physical activity, and smoking prevalence can vary significantly across racial and ethnic groups, contributing to the observed differences in mortality rates. For instance, smoking has been identified as a major risk factor for heart disease, with studies indicating that smokers have a significantly higher risk of dying from heart disease compared to non-smokers [25]. Furthermore, the role of neuroticism and psychological factors has been highlighted, with states exhibiting higher neuroticism correlating with elevated mortality rates from heart disease [25], suggesting that mental health may also play a critical role in these disparities.

The states with the highest AAMR percentiles, including Oklahoma, California, Tennessee, and West Virginia, may reflect regional differences in healthcare access, lifestyle, and environmental factors. The high AAMR in these states may be linked to factors like limited healthcare access, high prevalence of risk factors, and socioeconomic disparities [26,27]. Additionally, lifestyle and environmental influences in these regions could contribute to a higher disease burden. These factors combined may lead to elevated mortality rates from Alzheimer's and inflammatory heart disease.

To address these disparities, targeted public health interventions are necessary. Strategies could include enhancing access to healthcare services, promoting healthy lifestyle choices through community programs, and increasing awareness of the risk factors associated with both Alzheimer's disease and heart disease. Incorporating the E(e)SEEDi approach-focusing on Environment, Sleep, Emotion, Exercise, and Diet-could provide a holistic framework for promoting healthier lifestyles and preventing chronic diseases [28]. Additionally, culturally tailored educational initiatives could improve health literacy and encourage preventive behaviors in high-risk populations. Furthermore, integrating mental health support into chronic disease management could mitigate some of the psychological factors contributing to these health disparities [29,30].

## 5 Limitations

Our research has several constraints. The retrospective design limits our ability to ensure data quality and introduces potential biases associated with analyzing pre-existing data. Dependence on ICD codes may result in misclassification or underreporting of Alzheimer or IHD as a cause of death, and changes in coding practices over time may impact the consistency of trends. Furthermore, the lack of clinical information, such as disease stage or treatment history, as well as unaccounted confounding factors like socioeconomic status and access to healthcare, restricts the depth of our analysis. Moreover, the study cannot determine causality, and the applicability of our findings may be limited by potential reporting biases and regional differences in healthcare quality.

## 6 Conclusion

In conclusion, the decline in AAMR due to Alzheimer's disease and IHD from 1999 to 2020 reflects advances in medical treatments, prevention efforts, demographic shifts, and healthcare access improvements. However, persistent disparities across genders, races, and states underscore the need for continued research into biological, sociocultural, genetic, and socioeconomic factors, as well as targeted, multifaceted public health strategies. Future studies should explore the mechanisms driving these trends and evaluate interventions aimed at reducing mortality rates and promoting health equity across all populations.

## CRediT authorship contribution statement

**Muzamil Akhtar:** Writing – original draft, Formal analysis, Data curation, Conceptualization. **Hanzala Ahmed Farooqi:** Writing – original draft, Data curation. **Rayyan Nabi:** Writing – original draft. **Javed Iqbal:** Writing – original draft. **Sabahat Ul Ain Munir Abbasi:** Writing – original draft. **Muhammad Rashid:** Writing – review & editing. **Syed Khurram Mushtaq Gardezi:** Writing – review & editing. **David P. Ripley:** Writing – review & editing. **Raheel Ahmed:** Writing – review & editing.

## Ethics approval and consent to participate

Not Applicable.

## Consent for publication

Not Applicable.

## Availability of data and material

Data used to conduct analysis is given in supplementary file. Data not provided will be provided upon reasonable request to corresponding author.

## Data availability statement

Data used for this study is publicly available at https://wonder.cdc.gov.

## Funding

This research received no specific grant from any funding agency in the public, commercial, or not-for-profit sectors.

## Declaration of competing interest

The authors declare that they have no competing interests.
