# Supplementary material for: Trends in mortality due to ischemic heart diseases among patients with Alzheimer's disease in the United States from 1999 to 2020
Source: Int J Cardiol Cardiovasc Risk Prev. 2025 Mar 7;25:200390. doi: 10.1016/j.ijcrp.2025.200390 (PMC11929933; doi:10.1016/j.ijcrp.2025.200390)
Supplement: Multimedia component 1 [file mmc1.docx]

**Supplementary File:**

| **Table 1:** Frequency and age adjusted mortality rates per 100,000 deaths in adults aged 45+ in the United States, 1999 to 2020 | | | |
| --- | --- | --- | --- |
|  | **Deaths** | **Population** | **Overall AAMR per 100,000 deaths (95% CI)** |
| Entire Cohort | 171080 | 2622477732 | 6.66 (6.63 - 0.02) |
| ***Sex*** |  |  |  |
| Male | 59544 | 1226819075 | 6.36 (6.31 - 6.42) |
| Female | 111536 | 1395658657 | 6.79 (6.75 - 6.83) |
| **Census region of United States** |  |  |  |
| Northeast | 34369 | 497982485 | 6.41 (6.35 - 6.48) |
| Midwest | 37495 | 580373620 | 6.26 (6.2 - 6.33) |
| South | 56671 | 966355890 | 6.33 (6.27 - 6.38) |
| West | 42545 | 577765737 | 7.91 (7.83 - 7.98) |
| **Race** |  |  |  |
| NH Asian or Pacific Islander | 3002 | 117961848 | 3.65 (3.52 - 3.78) |
| NH Black or African American | 12715 | 275481172 | 6.49 (6.38 - 6.61) |
| NH White | 145563 | 1958646230 | 6.84 (6.81 - 6.88) |
| NH American Indian or Alaska Native | 408 | 17576489 | 4.08 (3.68 - 4.48) |
| Hispanic | 9037 | 252811993 | 5.93 (5.81 - 6.05) |

**Supplementary Table 1:** Frequency and age adjusted mortality rates per 100,000 deaths in adults aged 45+ in the United States, 1999 to 2020

| **Table 2:** Annual age adjusted mortality rates per 100,000 deaths in adults aged 45+ in the United States, 1999 to 2020 | |
| --- | --- |
| **Year** | **Age Adjusted Mortality Rate per 100,000 deaths (95% CI)** |
| 1999 | 10.56 (10.35 - 0.11) |
| 2000 | 10.42 (10.21 - 0.1) |
| 2001 | 10.43 (10.23 - 0.1) |
| 2002 | 10.61 (10.41 - 0.1) |
| 2003 | 10.11 (9.92 - 0.1) |
| 2004 | 9.79 (9.6 - 0.1) |
| 2005 | 9.32 (9.13 - 0.09) |
| 2006 | 8.68 (8.5 - 0.09) |
| 2007 | 8.08 (7.92 - 0.09) |
| 2008 | 7.45 (7.29 - 0.08) |
| 2009 | 6.83 (6.68 - 0.08) |
| 2010 | 6.46 (6.31 - 0.07) |
| 2011 | 5.79 (5.65 - 0.07) |
| 2012 | 5.23 (5.1 - 0.07) |
| 2013 | 4.95 (4.83 - 0.06) |
| 2014 | 4.4 (4.28 - 0.06) |
| 2015 | 4.42 (4.31 - 0.06) |
| 2016 | 4.34 (4.22 - 0.06) |
| 2017 | 4.1 (4 - 0.06) |
| 2018 | 3.97 (3.87 - 0.05) |
| 2019 | 3.74 (3.63 - 0.05) |
| 2020 | 4.03 (3.93 - 0.05) |

**Supplementary Table 2:** Annual age adjusted mortality rates per 100,000 deaths in adults aged 45+ in the United States, 1999 to 2020

| **Table 3:** Age adjusted mortality rates per 100,000 deaths stratified by sex in adults aged 65+ in the United States, 1999 to 2020 | | |
| --- | --- | --- |
|  | **Age Adjusted Mortality Rate per 100,000 deaths (95% CI)** | |
| **Year** | **Female** | **Male** |
| 1999 | 10.82 (10.56 - 11.08) | 9.82 (9.47 - 10.17) |
| 2000 | 10.73 (10.47 - 10.98) | 9.66 (9.32 - 10) |
| 2001 | 10.69 (10.44 - 10.94) | 9.77 (9.43 - 10.11) |
| 2002 | 10.9 (10.65 - 11.16) | 9.83 (9.49 - 10.17) |
| 2003 | 10.44 (10.19 - 10.68) | 9.37 (9.04 - 9.69) |
| 2004 | 9.94 (9.7 - 10.18) | 9.41 (9.09 - 9.74) |
| 2005 | 9.52 (9.29 - 9.75) | 8.8 (8.49 - 9.11) |
| 2006 | 8.76 (8.54 - 8.98) | 8.45 (8.15 - 8.75) |
| 2007 | 8.17 (7.96 - 8.38) | 7.78 (7.5 - 8.06) |
| 2008 | 7.68 (7.47 - 7.88) | 7.03 (6.77 - 7.3) |
| 2009 | 6.99 (6.79 - 7.18) | 6.51 (6.27 - 6.76) |
| 2010 | 6.41 (6.23 - 6.6) | 6.51 (6.26 - 6.75) |
| 2011 | 5.78 (5.61 - 5.96) | 5.77 (5.55 - 6) |
| 2012 | 5.19 (5.03 - 5.36) | 5.23 (5.02 - 5.45) |
| 2013 | 4.85 (4.7 - 5.01) | 5.02 (4.82 - 5.23) |
| 2014 | 4.36 (4.22 - 4.51) | 4.4 (4.21 - 4.59) |
| 2015 | 4.36 (4.22 - 4.51) | 4.48 (4.3 - 4.67) |
| 2016 | 4.2 (4.05 - 4.34) | 4.53 (4.34 - 4.72) |
| 2017 | 3.99 (3.86 - 4.13) | 4.25 (4.07 - 4.43) |
| 2018 | 3.9 (3.76 - 4.03) | 4.05 (3.88 - 4.22) |
| 2019 | 3.66 (3.53 - 3.79) | 3.79 (3.62 - 3.95) |
| 2020 | 3.92 (3.79 - 4.05) | 4.16 (3.99 - 4.33) |

**Supplementary Table 3:** Age adjusted mortality rates per 100,000 deaths stratified by sex in adults aged 65+ in the United States, 1999 to 2020

| **Table 4:** Age adjusted mortality rates per 100,000 deaths stratified by race in adults aged 45+ in the United States, 1999 to 2020 | | | | |
| --- | --- | --- | --- | --- |
|  | **Age Adjusted Mortality Rate per 100,000 deaths (95% CI)** | | | |
| **Year** | **NH Asian or Pacific Islander** | **Black or African American** | **NH White** | **Hispanic or Latino** |
| 1999 | 5.15 (4.08 - 6.42) | 10.36 (9.61 - 11.11) | 10.8 (10.57 - 11.02) | 7.68 (6.76 - 8.61) |
| 2000 | 4.85 (3.86 - 6.01) | 10.2 (9.46 - 10.94) | 10.65 (10.42 - 10.87) | 8.18 (7.25 - 9.11) |
| 2001 | 5.41 (4.34 - 6.48) | 9.75 (9.03 - 10.47) | 10.68 (10.46 - 10.9) | 8.68 (7.75 - 9.6) |
| 2002 | 4.54 (3.64 - 5.6) | 10.28 (9.54 - 11.02) | 10.86 (10.64 - 11.08) | 8.98 (8.05 - 9.91) |
| 2003 | 5.23 (4.24 - 6.22) | 9.94 (9.21 - 10.67) | 10.33 (10.12 - 10.55) | 8.3 (7.43 - 9.18) |
| 2004 | 4.44 (3.56 - 5.31) | 10.02 (9.3 - 10.75) | 9.89 (9.68 - 10.1) | 10.26 (9.31 - 11.2) |
| 2005 | 4.48 (3.64 - 5.33) | 9.98 (9.26 - 10.69) | 9.42 (9.21 - 9.62) | 8.82 (7.98 - 9.66) |
| 2006 | 4.14 (3.36 - 4.92) | 9.43 (8.74 - 10.12) | 8.75 (8.56 - 8.94) | 8.35 (7.55 - 9.14) |
| 2007 | 4.57 (3.77 - 5.37) | 8 (7.37 - 8.62) | 8.22 (8.04 - 8.41) | 7.73 (6.99 - 8.48) |
| 2008 | 5.09 (4.28 - 5.9) | 7.66 (7.06 - 8.27) | 7.53 (7.36 - 7.71) | 7.06 (6.36 - 7.75) |
| 2009 | 4.03 (3.32 - 4.73) | 7.16 (6.59 - 7.74) | 6.89 (6.72 - 7.06) | 6.68 (6.03 - 7.33) |
| 2010 | 4.49 (3.77 - 5.2) | 6.13 (5.6 - 6.65) | 6.56 (6.4 - 6.72) | 6.3 (5.69 - 6.92) |
| 2011 | 4.57 (3.89 - 5.26) | 5.81 (5.3 - 6.31) | 5.86 (5.71 - 6.02) | 5.75 (5.18 - 6.31) |
| 2012 | 4.04 (3.41 - 4.66) | 5.04 (4.59 - 5.5) | 5.26 (5.12 - 5.4) | 5.37 (4.84 - 5.9) |
| 2013 | 3.71 (3.14 - 4.28) | 4.63 (4.2 - 5.07) | 4.95 (4.82 - 5.09) | 5.7 (5.17 - 6.22) |
| 2014 | 3.08 (2.58 - 3.58) | 4.17 (3.77 - 4.58) | 4.4 (4.27 - 4.53) | 5.1 (4.62 - 5.58) |
| 2015 | 2.93 (2.46 - 3.4) | 4.24 (3.84 - 4.64) | 4.47 (4.34 - 4.59) | 4.64 (4.2 - 5.08) |
| 2016 | 3.1 (2.64 - 3.57) | 4.37 (3.97 - 4.77) | 4.34 (4.21 - 4.46) | 4.7 (4.27 - 5.13) |
| 2017 | 2.99 (2.55 - 3.43) | 4 (3.63 - 4.38) | 4.14 (4.02 - 4.26) | 4.13 (3.74 - 4.52) |
| 2018 | 2.54 (2.14 - 2.93) | 3.5 (3.15 - 3.85) | 4.1 (3.98 - 4.22) | 4.08 (3.7 - 4.46) |
| 2019 | 2.2 (1.85 - 2.56) | 3.52 (3.18 - 3.86) | 3.8 (3.68 - 3.91) | 4.03 (3.66 - 4.41) |
| 2020 | 2.93 (2.53 - 3.33) | 4.03 (3.67 - 4.39) | 4.08 (3.96 - 4.2) | 4.24 (3.86 - 4.61) |

**Supplementary Table 4:** Age adjusted mortality rates per 100,000 deaths stratified by race in adults aged 45+ in the United States, 1999 to 2020

| **Table 5:** Age adjusted mortality rates per 100,000 deaths stratified by census region in adults aged 45+ in the United States, 1999 to 2020 | | | | |
| --- | --- | --- | --- | --- |
|  | **Age Adjusted Mortality Rate per 100,000 deaths (95% CI)** | | | |
| **Year** | **Northeast** | **Midwest** | **South** | **West** |
| 1999 | 10.74 (10.29 - 11.18) | 9.61 (9.21 - 10.01) | 10.55 (10.19 - 10.9) | 11.67 (11.17 - 12.17) |
| 2000 | 10.38 (9.94 - 10.82) | 9.81 (9.41 - 10.21) | 10.04 (9.7 - 10.39) | 11.88 (11.38 - 12.38) |
| 2001 | 10.15 (9.72 - 10.58) | 9.39 (9 - 9.78) | 10.29 (9.95 - 10.64) | 12.33 (11.83 - 12.83) |
| 2002 | 10.38 (9.95 - 10.81) | 9.34 (8.95 - 9.72) | 10.74 (10.39 - 11.09) | 12.18 (11.69 - 12.67) |
| 2003 | 9.63 (9.21 - 10.04) | 9.11 (8.73 - 9.49) | 10.02 (9.69 - 10.36) | 12.02 (11.53 - 12.5) |
| 2004 | 9.35 (8.95 - 9.75) | 8.68 (8.32 - 9.05) | 9.67 (9.34 - 9.99) | 11.85 (11.37 - 12.32) |
| 2005 | 8.72 (8.33 - 9.1) | 8.53 (8.17 - 8.89) | 9.2 (8.88 - 9.51) | 11.07 (10.62 - 11.52) |
| 2006 | 7.74 (7.38 - 8.09) | 7.91 (7.56 - 8.25) | 8.53 (8.23 - 8.83) | 10.82 (10.38 - 11.26) |
| 2007 | 7.51 (7.16 - 7.86) | 7.37 (7.04 - 7.7) | 7.88 (7.6 - 8.17) | 9.85 (9.44 - 10.26) |
| 2008 | 6.72 (6.39 - 7.05) | 6.91 (6.6 - 7.23) | 7.15 (6.88 - 7.41) | 9.35 (8.95 - 9.74) |
| 2009 | 6.34 (6.02 - 6.66) | 6.19 (5.89 - 6.49) | 6.8 (6.54 - 7.06) | 8.04 (7.68 - 8.4) |
| 2010 | 5.99 (5.68 - 6.3) | 5.64 (5.36 - 5.92) | 6.42 (6.17 - 6.67) | 7.86 (7.51 - 8.21) |
| 2011 | 5.16 (4.88 - 5.45) | 5.37 (5.1 - 5.64) | 5.3 (5.07 - 5.52) | 7.59 (7.25 - 7.93) |
| 2012 | 5.22 (4.93 - 5.5) | 4.76 (4.5 - 5.02) | 4.74 (4.53 - 4.95) | 6.53 (6.21 - 6.84) |
| 2013 | 4.62 (4.36 - 4.89) | 4.71 (4.45 - 4.96) | 4.43 (4.24 - 4.63) | 6.35 (6.05 - 6.65) |
| 2014 | 3.92 (3.68 - 4.17) | 4.26 (4.02 - 4.5) | 4.03 (3.84 - 4.21) | 5.55 (5.28 - 5.83) |
| 2015 | 4.06 (3.82 - 4.31) | 3.91 (3.69 - 4.14) | 4.15 (3.96 - 4.34) | 5.71 (5.43 - 5.99) |
| 2016 | 4.17 (3.92 - 4.42) | 4.13 (3.9 - 4.36) | 3.85 (3.67 - 4.03) | 5.44 (5.17 - 5.71) |
| 2017 | 3.87 (3.63 - 4.1) | 3.97 (3.74 - 4.19) | 3.75 (3.58 - 3.92) | 5 (4.74 - 5.26) |
| 2018 | 3.73 (3.5 - 3.96) | 4.26 (4.03 - 4.49) | 3.51 (3.35 - 3.68) | 4.62 (4.38 - 4.87) |
| 2019 | 3.47 (3.25 - 3.69) | 3.79 (3.57 - 4) | 3.41 (3.26 - 3.57) | 4.4 (4.16 - 4.63) |
| 2020 | 3.83 (3.6 - 4.06) | 4.34 (4.11 - 4.57) | 3.63 (3.47 - 3.8) | 4.55 (4.32 - 4.79) |

**Supplementary Table 5:** Age adjusted mortality rates per 100,000 deaths stratified by census region in adults aged 45+ in the United States, 1999 to 2020

| **Table 6:** State wise age adjusted mortality rates per 100,000 deaths in adults aged 45+ in the United States, 1999 to 2020 | | |
| --- | --- | --- |
| **State** | **AAMR per 100,000 deaths (95% CI)** | **Percentage of Deaths** |
| Alabama | 5.96 (5.71 - 6.21) | 1.32% |
| Alaska | 3.2 (2.53 - 3.98) | 0.05% |
| Arizona | 5.52 (5.32 - 5.73) | 1.68% |
| Arkansas | 6.48 (6.16 - 6.79) | 0.95% |
| California | 10.5 (10.37 - 10.62) | 16.98% |
| Colorado | 4.48 (4.26 - 4.71) | 0.87% |
| Connecticut | 5.9 (5.65 - 6.15) | 1.26% |
| Delaware | 7.11 (6.51 - 7.71) | 0.32% |
| District of Columbia | 9.15 (8.28 - 10.02) | 0.25% |
| Florida | 4.89 (4.8 - 4.99) | 6.08% |
| Georgia | 4.96 (4.78 - 5.14) | 1.67% |
| Hawaii | 4.23 (3.88 - 4.58) | 0.34% |
| Idaho | 4.39 (4.01 - 4.77) | 0.30% |
| Illinois | 5.5 (5.36 - 5.64) | 3.44% |
| Indiana | 7.15 (6.92 - 7.38) | 2.22% |
| Iowa | 7.67 (7.37 - 7.98) | 1.48% |
| Kansas | 4.54 (4.29 - 4.8) | 0.70% |
| Kentucky | 6.8 (6.52 - 7.08) | 1.34% |
| Louisiana | 5.29 (5.04 - 5.54) | 1.04% |
| Maine | 5.09 (4.71 - 5.47) | 0.40% |
| Maryland | 5.92 (5.69 - 6.14) | 1.55% |
| Massachusetts | 4.19 (4.03 - 4.35) | 1.58% |
| Michigan | 6.92 (6.74 - 7.09) | 3.51% |
| Minnesota | 3.43 (3.26 - 3.6) | 0.96% |
| Mississippi | 8.64 (8.26 - 9.03) | 1.14% |
| Missouri | 7.22 (6.99 - 7.45) | 2.26% |
| Montana | 3.46 (3.08 - 3.84) | 0.19% |
| Nebraska | 5.08 (4.75 - 5.42) | 0.52% |
| Nevada | 3.66 (3.37 - 3.95) | 0.35% |
| New Hampshire | 6.1 (5.64 - 6.55) | 0.41% |
| New Jersey | 7.35 (7.16 - 7.53) | 3.44% |
| New Mexico | 5.52 (5.15 - 5.89) | 0.51% |
| New York | 6.92 (6.8 - 7.05) | 7.21% |
| North Carolina | 6.09 (5.91 - 6.27) | 2.56% |
| North Dakota | 8.02 (7.37 - 8.68) | 0.35% |
| Ohio | 7.52 (7.36 - 7.69) | 4.61% |
| Oklahoma | 10.99 (10.61 - 11.36) | 1.96% |
| Oregon | 4.31 (4.09 - 4.54) | 0.87% |
| Pennsylvania | 6.32 (6.19 - 6.46) | 5.02% |
| Rhode Island | 7.46 (6.95 - 7.96) | 0.50% |
| South Carolina | 6.01 (5.75 - 6.27) | 1.24% |
| South Dakota | 7.43 (6.84 - 8.02) | 0.37% |
| Tennessee | 10.32 (10.03 - 10.6) | 2.97% |
| Texas | 6.9 (6.77 - 7.04) | 6.18% |
| Utah | 3.18 (2.89 - 3.47) | 0.28% |
| Vermont | 7.75 (7.03 - 8.47) | 0.26% |
| Virginia | 4.64 (4.46 - 4.81) | 1.57% |
| Washington | 7.61 (7.38 - 7.85) | 2.34% |
| West Virginia | 9.67 (9.21 - 10.13) | 1.00% |
| Wisconsin | 4.86 (4.67 - 5.05) | 1.50% |
| Wyoming | 4.66 (4.01 - 5.32) | 0.11% |
| Alabama | 5.96 (5.71 - 6.21) | 1.32% |

**Supplementary Table 6:** State wise age adjusted mortality rates per 100,000 deaths in adults aged 45+ in the United States, 1999 to 2020
